# The publicness enigma: Can perceived publicness predict employees’ formal and prosocial behavior across sectors?

**DOI:** 10.1371/journal.pone.0262253

**Published:** 2022-02-10

**Authors:** Yinnon Dryzin-Amit, Dana R. Vashdi, Eran Vigoda-Gadot

**Affiliations:** Division of Public Administration and Policy, School of Political Sciences, University of Haifa, Haifa, Israel; Taiyuan University of Science and Technology, CHINA

## Abstract

The meaning of Publicness for organizations and for individuals has received growing attention in the public administration literature in recent years. We adopt a perceived publicness perspective to expand our understanding of the publicness concept and operationalize this perception as a means to predict employees’ formal and prosocial behaviors across sectors. Using a recently developed Publicness Perceptions Scale (PPS), we present and empirically examine a model regarding the direct and indirect relationships among perceived publicness, employees’ engagement, and their performance in public and hybrid organizations. Findings based on a field study of 340 employees from governmental (i.e. public) and non-governmental (i.e. hybrid) organizations reveal that perceived publicness has a positive relationship with Organizational Citizenship Behaviors (OCB) and that this relationship is largely mediated by employees’ engagement. In addition, in non-government organizations perceived publicness is negatively related to employees’ in-role performance. We thus contribute to the theoretical knowledge on publicness at the perceptual level and point to its role in formal and informal performance across sectors. Other theoretical, methodological, and practical implications are discussed, and directions for future studies are suggested.

## Introduction

The meaning of publicness in social science is under dispute and in many ways enigmatic. It reflects an ongoing debate about normative limits and borders of the public and private realms (e.g., [1–3], to name only few). To date, studies offer a varied mixture of concepts aimed at capturing the meaning of publicness within and between the private and public sectors (e.g., [4]) and for individuals as employees, clients or as citizens (e.g., [5]). Most studies use a dimensional approach to publicness, theorizing that ownership (public, private, or nonprofit), funding (government grants versus consumer payments), and control (by political versus market forces) are at the heart of the distinction between public and private organizations [5, 6]. Recently this approach is challenged by alternative perspectives and empirical examinations arguing for a broader meaning of publicness (e.g., [4, 6–9]). Critical questions emerge in light of this dispute. Should governments define the public sphere and public interest in a broader or narrower way? How may such definitions affect organizations, employees, clients, and citizens? What is the meaning of the distinction between private and public for the economy and for society as a whole? How are nations, organizations, and individuals affected by the separation of the public from the private, and what is the impact of such distinctions on organizational outcomes and on human behavior [10, 11]?

Our study maintains that one way to enhance our understanding of what publicness means is by examining the perceptions of publicness employees have in regards to their organization. Essentially, we suggest that publicness is the individual’s perception of *“the extent of attentiveness* [of all organizations] *to the* [interests of the] *public”* [6]. Several factors may affect this perception, including the organization’s ownership, source of funding, and control through political authority or the market [5] as well as the organization’s values [7]. We rely on previous research showing relationships between engagement, and both in-role performance and OCB (e.g., [12, 13]), and maintain that employees, who perceive their organization as high on a publicness scale are likely to gain meaning and a sense of purpose, as well as see their job as an opportunity to realize aspired values [14]. As a result, they are likely to be more engaged and more willing to perform their job as well as perform in a manner that goes beyond their job expectations (i.e. OCB behaviors; [15, 16]). The theoretical framework of the current study is based on the New Institutionalism Theory in political science and public administration. We rely specifically on the works of March and Olsen [17, 18], DiMaggio and Powell [19], and Moulton [7] who emphasize the role of symbols and values in developing people’s perceptions about and attitudes towards society.

To test the relationships proposed above we take three main steps. First, we try to suggest a common meaning of publicness in various types of organizations (e.g., private, public, hybrid) and for different types of employees (e.g., line employees, public servants, and private employees), and to confirm that it aligns with existing knowledge (e.g., [20–24]). Our definition proposes that the way people rank their social environment, organization, and the types of goods and services they provide on a publicness scale might have a significant effect on their behavior in the workplace. Second, we use a recently developed and validated perceived publicness scale (PPS) [25] to propose a model and a set of hypotheses connecting perceptions of publicness with performance (i.e., in-role performance and organizational citizenship behavior [OCB]) across organizations, both directly and indirectly through employees’ engagement (i.e., EE) and taking organizational ownership into consideration. Finally, our model and hypotheses are empirically tested using a sample of 340 employees from governmental and non-governmental organizations. Our findings, which largely support the model, are used to illustrate the advantages of the perceptual approach towards publicness.

The paper proceeds as follows: We start with an overview of the major conceptual developments in publicness theory, moving from grand thinking on the meaning of publicness to what has been discussed in the public administration and organizational literature regarding the distinction between private and public organizations. We then introduce our theoretical framework based on neo-institutional ideas suggesting our model and hypotheses. This is followed by a comprehensive presentation of our methods and design, the empirical finings and finally a discussion with theoretical and practical conclusions and implications.

## Theoretical background

### Grand thinking on the meaning of publicness

What is the meaning of publicness and is there a clear distinction between the “public” and the "private" in the scientific thinking and literature? Previous studies struggle with these questions, usually adopting either an interdisciplinary approach (e.g., [11]) or conducting a cross-sector comparison (e.g., [26]). According to Weintraub and Kumar [11] social, cultural, and political theories may help distinguish what is public from what is private taking into consideration their variation across context, time, and space. More specifically, Weintraub [10] suggests a taxonomy by which households, domesticity, family, and the market economy belong to the private realm whereas political community, sociability, and government administrative interventions belong to the public realm. In contrast, [27] is much more straightforward by stating that *“the private is what the public is not”* and suggesting that the standing of the private realm evolves mainly *“due to the increasing difficulties of the public realm in achieving its own standards for the control and management of the accelerating internationalization of intractable social problems*.*”*

Another perspective views publicness as part of the overall public service ethos. As such it is often considered a collective domain, distinguished from the private domain by highlighting several major principles such as equity, accountability, representation, openness, impartiality, justice and responsiveness to citizens’ needs and expectations (e.g., [28–30]). Succinctly, one can view publicness as *“the extent of attentiveness to the public”* ([6]; 471). In recent decades, the neo-liberal public administration spirit has posed a strong challenge to this notion of publicness. As a result, the traditional public sphere-oriented jargon is gradually being replaced by alternative ideas from the private sector such as market-driven reforms, business-like norms and values, competitiveness, clientism, efficiency, productivity, and profitability [30]. These later principles sharply contradict the private ones mentioned by Weintraub [10] and [27]. Hence, the meaning of publicness today is defined more and more as an antidote to neo-liberalism and the rule of the markets (e.g., [7, 8]). This change reflects the need to redefine publicness in a more coherent manner that can also help us explain and understand performance in these sectors [31, 32].

The literature suggests several specific definitions for publicness that may also help differentiate between public and private sectors and/or organizations. Rainey [33] and Bozeman and Bretschneider [22] capture the essence of the "public" based on two main principles [11, 23] which represent the production of goods and the influence of organizations on governments. Production of goods is based on the economic approach whereas the way organizations influence the government and society is based on a normative approach [34, 35]. In other words, public sector organizations are theoretically distinguished from private ones in the way they affect the public’s interests or are involved in the production of public goods. Benn and Gaus [36] recognized these two principles, suggesting that they are located at the opposite poles of a publicness continuum and tried to reconcile them. According to their main arguments, the first pole focuses on “public interest” and relies on the political theory of liberalism. The second comprises the organic perspective of publicness and focuses on the production of “public goods.”

The political liberalism theory sees fundamental autonomy of the individual as his/her right which should not be to be interfered with by others [23]. According to this philosophy, any good that is not private naturally becomes public. Individuals need to interact with others in order to help provide their daily necessities, and conflicts frequently arise during such interactions. Consequently, a political sphere, derived from the autonomy of individuals, was established in order to resolve disputes [37]. Therefore, according to political liberalism, the essence of publicness is not to produce goods and services but to determine which of them are in the public’s interest and then regulate their production. Governments have tended to adopt this approach, making the concept of publicness increasingly relevant to private organizations that deal with the production of public goods [38, 39].

In contrast to the political liberalism approach the organic perspective argue that individuals are not considered to be fully autonomous beings, but rather part of a community that endows them with characteristics which are public in nature. From this viewpoint, publicness does not create a dichotomous distinction between public and private. The public sphere encompasses the entirety of social life and includes the private sphere. The state was established to provide structure and coordination to the public sphere. This approach is evident mostly in totalitarian political systems in which the interests of the state precede the interests of the individual [40]. In democracies, elements of the organic way of thinking are also present, in particular in the requirement of citizen involvement and participation in state decision making [41, 42].

All in all, several dimensions are commonly used in the literature to explore the meaning of publicness as a scholarly concept: (1) the relations to the state (e.g., ownership), (2) the level of concern to everyone (e.g., public funding), (3) the level of accessibility to everyone, and (4) the aim for a common good or shared interest (e.g., public values) [11, 43]. The meaning of "private" can be easily understood by using the antonyms. However, the meaning of "private" also includes pertaining to private property in a market economy (e.g., [8]). Yet, the question of the contribution of publicness to performance has, as far as we can tell, failed to consider all aspects of this concept in a comprehensive and rigorous empirical research.

### Publicness in organizational and administrative studies

Bozeman [5] suggested that all organizations are public. However, do all organizations and employees consider publicness in the same way? Do we all mean the same thing when we tag an organization, service, or resources as "public"? What does publicness mean in the eyes of those who produce goods and services in governmental and in non-governmental organizations, and how valid are their perceptions? Over the years, publicness in administrative and organizational science has acquired many different meanings depending on its context, time, and space [6]. In the public administration literature discussion on publicness is largely affected by the conceptual shift from government to governance (e.g., [44]) and by the evolvement of more hybrid organizational structures that blur the borders between private and public [28]. Today, publicness is frequently regarded as part of the public interest discourse representing the extent to which the target (e.g., the organization or nation) maximizes the collective benefit of government policies [36]. Within this literature, several approaches to publicness have evolved including the generic [45], legal ownership [22, 33], dimensional [5], and realized public-value [7] approaches. According to the *generic* approach, public and private organizations are viewed as not having meaningful differences in terms of management methods, decision-making processes, or organizational structures. Once general priorities are established, *“public and private bureaucracies operate the same”* ([45], p. 365). However, more recent studies have criticized the generic approach, contending that public organizations maintain distinct goals, structures, processes, and motivations [12, 46]. Alternatively, the *legal ownership* view (e.g., [22, 33]) suggests that publicness is subject to legal and ownership criteria. Due to their unique legal standing, public organizations face unique challenges such as complexity (resulting from heterogeneous types of stakeholders); permeability (resulting from the strong impact of external events); instability (resulting from political control, changes in policy, and constant pressure to achieve rapid results); and finally, the absence of competitive market related pressures. The *dimensional* approach [5] argues that publicness is captured by the degree to which an organization is subject to political, economic, and social constraints. Bozeman [5] suggested a two-dimensional empirical publicness grid classifying publicness on external economic and political authority axes. Accordingly, publicness is determined by the extent to which an institution or organization exercises or is constrained by political authority and is private to the extent that it exercises or is constrained by economic authority.

Moulton [7] integrated Bozeman’s [5, 8] theory of dimensional publicness with the *public values* theory, and presented a framework of “realized publicness,” defined as public outcomes that are in line with predetermined public values, achieved through public value institutions. Several later works have supported Moulton’s framework (e.g., [29, 47, 48]). In line with the realized publicness approach (e.g., [48–50]). Jørgensen and Bozeman [51] identified seven categories of public values: those dealing with the public sector’s contributions to society (e.g., altruism); the transformation of interests into decisions (e.g., democracy); the relationship between public administrators and politicians (e.g., accountability); the relationship between public administrators and their environment (e.g., responsiveness); intra-organizational aspects of public administration (e.g., reliability); the behavior of public-sector employees (e.g., integrity); and the relationship between public administration and citizens (e.g., equity). As we explain below, similar to the public values perspective, we too take a neo-institutional perspective when defining publicness. However, we propose that, given the influence of organizational symbols, values, and traditions on publicness, its definition is largely conceived in the minds of individuals and thus can be better captured by their perceptions about the concept.

### Perceived publicness and neo-institutionalism

As can be drawn from the review presented above, the organizational and administrative literature have failed to consider all aspects of the publicness concept in a comprehensive and rigorous empirical manner (e.g., [4, 9]). To advance knowledge on publicness the current study tries to take one-step forward, arguing that regardless of sector affiliation, all dimensions of publicness can be simultaneously examined by studying employees’ perceptions of the extent to which their organization is public. Such an examination of perceived publicness, which can include all aspects of publicness (even such that may objectively contradict each other such as market control and governmental ownership), may hold together in the mind of an individual [7, 50].

Perceived publicness builds on the assumption that individual behavior is driven by perceptions of reality, not reality itself [52]. Given that it is employees who provide public services at the ground level, it is important to understand the consequences of employees’ perceptions of publicness within public organizations, as well as in other organizations. The blurring of boundaries between the sectors means that perceptions of publicness may have consequences for non–public-sector organizations as well. Like in other studies, we use the neo-institutional theory [7, 17–19] as a theoretical foundation. This theory suggests that individuals cannot be separated from their social environments. Thus, symbols, rituals, beliefs, and values that structure political processes and administrative procedures influence the perceptions and behaviors of employees. It therefore emphasizes the mutual impact that organizations have on those working in them, and on the counter-impact of individuals’ perceptions and behaviors on the organizations [17, 18]. Based on the neo-institutional theory we assert that all of the dimensions of publicness (i.e., ownership, funding, control, and public values) may be aggregated into a coherent perceptual construct.

Two arguments are relevant to explaining this assertion. First, publicness reflects symbolic decisions and actions in institutions and may be reflected by those symbols. According to March and Olsen [17], institutions are full of symbols assuring all observers that events have occurred in the proper and most reliable way. As far as perceived publicness is concerned, employees subscribe to the values and attitudes of their institution. They do so due to not only the duties or obligations imposed on them by their roles within the organization, but also because institutions give meaning to the various activities they perform within their roles. Hence, perceived publicness provides an interpretation of life within the organization. It provides insights into the manner in which the employees understand their organization’s relationship with the political, social, and economic arenas, with communities, and with other organizations. Second, perceived publicness is self-sustaining. It gives people a sense of self-confidence about the meaning of their job and the raison d’etre for staying with the organization and investing effort in its growth and progress. As March and Olsen [18] maintain, *“Individuals organize arguments and information to create and sustain a belief in the wisdom of the action chosen*, *thus in the enthusiasm required to implement it”* (p. 40). In subscribing to the set of values included in perceived publicness, individuals may attach symbolic meaning to their role in the organization that justifies their responsibility, existence, and deeper meaning as employees, citizens, and clients. In all forms of meaning, it endows them with a sense of value and significance.

Thus, defining publicness as a perceptual construct has several advantages. First, it offers a theoretical explanation for why employees across all sectors hold various perceptions of publicness, regardless of the official sector of their organization [50]. Second, it offers a potential explanation for why employees from a diverse range of functional, professional, socio-economic, and educational backgrounds share a common set of public values within and across organizational contexts. Finally, it offers a potential explanation for the manner in which employees behave, especially when such behaviors do not sit comfortably with their private or personal interests or convictions [25]. Such explanations also shed light on the extent to which employees feel connected to their organization and are willing to invest in their jobs (i.e., employee engagement).

### The relationship between publicness and performance

Can publicness affect individual and organizational outcomes, and especially performance of employees in various worksites? Previous research has attempted to answer this question. First, an extensive meta-analysis by Boyne [9] suggested that the three dimensions of publicness—legal ownership, funding, and control—are not only conceptually but also empirically distinct from one another. Hence, all three dimensions need to be included when examining the effects of publicness on any dependent variable. However, a decade later, Andrews, Boyne, and Walker [4] reported that most publicness studies used only legal ownership or source of funding to proxy for publicness and its outcomes. In addition, they noted that, when examining organizational performance as a dependent variable based on these dimensions of publicness separately, the effect diminishes once differences in management and external factors are considered. In fact, they maintained that most of the 31 studies they reviewed focused on the effects of publicness-as-ownership on organizational efficiency and effectiveness. There are far fewer studies on publicness-as-funding and control, and even fewer have considered equity as a core element of publicness. Only two articles included measures of all three dimensions of publicness (i.e., [53, 54]). The effects of funding and control on organizational efficiency were almost all insignificant, and the impact of ownership on efficiency and effectiveness was evenly spread across positive, negative, and insignificant findings. The two strongest relationships found were between public ownership and organizational equity and between public funding and efficiency, but a rigorous examination of the data obtained in these two studies revealed great ambiguities [55]. Finally, political control, which is a very important measure of publicness [4, 7], was examined in only two studies reviewed by Andrews et al. [4]. Bartel and Harrison [53] showed that trade protection practices lead to poorer performance among Indonesian public corporations in the manufacturing industry. Similarly, Heinrich and Fournier [54] demonstrated that the effects of accreditation processes on substance abuse treatment programs in the United States were very small.

Another empirical approach to examining the consequences of the extent of publicness characterizing an organization relied on public values and the means to realize them, termed “normative publicness” (e.g., [49, 50]). Such research found that publicness is related to specific differences in sectorial values. For example, traditional public sector values reflect impartiality, incorruptibility, and lawfulness. On the other hand, traditional private sector values highlight profitability, innovation, and honesty. In addition, there are shared organizational norms and values between the sectors including accountability, expertise, reliability, effectiveness, and efficiency [50]. Yet none of these studies examined the relationship between these differences in norms and performance.

In view of the empirical fog and mixed findings on the meaning of publicness and on its relationship with employees’ performance, we suggest a specific model and set of hypotheses to illustrate the usefulness of the perceived publicness approach. We maintain that this approach may help capture various aspects of publicness, including factors that may objectively contradict each other, but that may hold together in the mind of an individual and help explain individual behaviors and organizational outcomes [7, 50].

## Model and hypotheses

### The relationship between perceived publicness, performance, and OCB

Two arguments based on the neo-institutional theory [17] can help in establishing the potential relationship between perceived publicness and employees’ performance. The first argument is directed towards intra-organizational relations and dynamics. The second is directed towards the extra-organizational environment, meaning, the consumers, clients, and citizens who benefit from the organizational outcomes.

First, perceived publicness reflects a symbolic system that teaches employees the right way to behave and perform inside the organization and in accordance with its values and culture. Employees may subscribe to the symbols embedded in the regulatory elements (e.g., laws and ownership), normative elements (e.g., standards) and cultural cognitive elements (e.g., public values) of publicness as part of their institutional identification with the organization. Thus, they will try to meet internal codes of behavior that are in line with internal solidarity and collective responsibility towards other co-workers. In doing so, they may behave with greater attention not only to the formal requirements related to the right way to act, but also to the informal codes of behavior about helping others or advancing the organization’s interests in general, beyond their formal duty. In doing so, they become good organizational citizens who care about the job and about fellow employees [56].

Second, higher levels of perceived publicness might be related not only to the duty or obligations imposed on employees by their internal roles within the organization, but also to seeing themselves as having a responsibility towards extra-organizational stakeholders. The neo-institutional approach maintains that organizations are socio-political institutions that also give external meaning to the various activities that employees perform within their roles [7]. According to this perspective, employees will become cognitively committed to meeting the symbolic messages delivered to them by the institution because they see themselves as having responsibilities towards external populations. A set of public-oriented symbols and norms that reflect overall responsibility to many others, beyond simply other fellow workers or even the limited audience of clients or customers, is a powerful signal of collective accountability to vast populations, many of them having no business-oriented or market-oriented type of exchange relations with the organization. Such a sense of accountability and responsibility may motivate employees to invest more resources in their job and improve their performance simply to meet the needs and expectations of external clients, customers, and citizens.

In view of this internal and external social responsibility, employees who regard their organization as favoring publicness will be more likely to have a greater sense of dedication, loyalty, and devotion, resulting in more investment in improving their formal and informal performance. They will try to maximize their own utility from their positions and behave according to the expectations that their perceived publicness defines for them. Hence, the institutional elements embedded in internal and external organizational socialization processes (i.e., through coercive, mimetic, and normative mechanisms) may help explain employees’ attitudes and behaviors towards others. Institutional factors affect how employees relate to many other stakeholders by emphasizing their collective responsibility to the public. In other words, higher levels of perceived publicness may encourage a duty to serve, a sense of responsibility to others, and mission-oriented attitudes and behavior that promote better in-role performance, reflected in better fulfillment of formal duties at work.

Therefore, we argue that perceived publicness might be related not only to behavior such as employees’ level of in-role performance, but also to their organizational citizenship behavior (i.e., OCB) through an institutional identification mechanism [17, 19]. Both in-role and prosocial/extra-role behaviors are crucial for achieving organizational goals [57]. Studies have demonstrated the positive relationship between prosocial behavior such as OCB and elements of publicness. For example, Bullock and his colleagues [12] suggested that employees rely on organizations to provide them with an opportunity to actualize their personal altruistic values. Employees deem it worthy to invest their physical, cognitive, and emotional energy to achieve the mission of the organization if they buy into its values [58]. Recently, Vogel and Willems [13] used an experimental approach to examine the outcomes of public servants’ awareness of the prosocial and societal impact of their actions. They used micro-interventions in the form of three preregistered experiments that emphasized making public service employees aware of their prosocial or societal impact. Their findings revealed that such awareness could have a positive effect on the employees’ well-being, intention to stay in the job, and willingness to recommend their respective jobs to others. Thus, when perceptions of publicness are high, the organization may become an essential vehicle for making a difference in society. In such cases, employees are more likely to bring their whole self to their work roles. They will go out of their way to fulfill their formal duties as well as assist other employees and engage in prosocial behaviors that benefit the organization and its goals. Accordingly, we expect to find a positive relationship between perceived publicness and employees’ behaviors such as in-role and prosocial behaviors/ OCB:

*H*_*1a*_: *Perceptions of publicness are positively related with employees’ in-role performance*.

*H*_*1b*_: *Perceptions of publicness are positively related with employees’ OCB*.

### The mediating role of employee engagement

Employee engagement represents a positive, affective, motivational, work-related state of mind characterized by vigor, absorption, and dedication [16, 58–60]. Two explanations offered to explain the relationship between employee engagement and other variables are the “broaden and build” theory of positive emotions [61] and the “conservation of resources” theory [14]. The broaden and build theory suggests that positive affect broadens the frequency and quality of associations between thoughts and actions and builds personal resources. The conservation of resources theory suggests that employees seek to protect, retain, and accumulate resources in order to realize their goals and improve their wellbeing.

Kahn’s [58] original conceptualization of engagement and Saks`s [60] multi-dimensional approach to employee engagement led other studies to examine differences in employee engagement levels across sectors. Vigoda-Gadot, Eldor, and Schohat [62] reported that employee engagement is higher among public sector employees than private sector employees. This finding is in line with other studies showing that public sector employees are motivated by a sense of mission and prefer intrinsic rewards [12]. Such studies also point to the crucial role of person-organization fit and value congruence in building engagement and promoting organizational success [63]. Accordingly, meaningfulness of the self is a critical variable that promotes value congruence and improves engagement and performance in organizations. The centrality of values means that employees rely on organizations to provide an opportunity for them to actualize their personal altruistic values [12]. Employees deem it worthy to invest their physical, cognitive, and emotional energy in the mission of the organization if they buy into its values. This contention accords with the neo-institutional theory that highlights the meaning of symbols, norms, and values in connecting individuals to the workplace. Thus, in some respects, public-oriented organizations become an essential vehicle in making a difference in society. Employees who strongly identify with the perceived publicness-oriented values in the organization may become more engaged in their work. Such an engagement is reflected in their greater willingness to bring their whole self to their work roles and associate their self-image with the job and the organization. As a result, employees will be more likely to have more psychological attachment to their jobs and to the organization in general [58].

Therefore, we argue that perceived publicness may be related to employees’ performance indirectly through their engagement. Strong perceptions about publicness may promote more engagement. Employees who think that there is potential to benefit society through attentiveness to the public interest [6] are likely to feel more vigor, absorption in and dedication to their job and organization. Such perceptions are likely to increase their feelings of worth, feelings that life has meaning and purpose, a sense of pride, hope, and an overall positive feeling about one`s role in society. These positive emotions broaden an employee`s thoughts, actions, and inspiration and build personal resources, ranging from physical and intellectual resources to social and psychological resources [61]. These resources, in turn, enhance the employees’ ability to perform their jobs and provide the resources to undertake prosocial and extra-role behaviors. In sum, perceptions that the employees’ organizations are committed to publicness are likely to have a positive effect on them, increasing their engagement. Engaged employees are more likely to take advantage of their current job resources and create new ones [64]. Doing so may lead to positive organizational outcomes in the form of better formal and informal performance [12, 15, 16, 65]. Thus, we hypothesize that:

*H*_*2*_: *Employees’ engagement mediates the relationship between publicness perceptions*, *and in-role and OCB performance*.

### Publicness, engagement, and performance across sectors: A moderating effect

We also examined the possible moderating effect of organizational ownership (e.g., governmental and non-governmental) on the relationship between perceived publicness and employees’ performance. Based on cross-sectorial studies on publicness, engagement, and performance (e.g., [33, 62, 66]), we argue that the institutional processes typical of government-owned organizations will strengthen this relationship for both in-role and prosocial extra-role behaviors such as OCB. In contrast, non-governmental (private or hybrid) organizations are not expected to serve society as such, but to do so only through their actions in the marketplace. Therefore, we expect the relationship between perceived publicness and performance will be much more modest than in government-owned organizations. For-profit entities are supposed to manage their operations efficiently to maximize their returns and meet the needs of their customers. Therefore, perceptions of publicness are only partially relevant to them. In fact, strong perceptions about publicness in private or hybrid organizations may actually reinforce value dissonance if they contradict the primary goals of these organizations. Hence, at the individual level, we would expect a stronger relationship between perceived publicness and the performance (in-role and prosocial/extra-role behaviors such as OCB) of governmental employees, as compared with the same relationship for non-governmental employees. Therefore, we hypothesize that:

*H*_*3*_: *Organizational ownership moderates the relationship between publicness perceptions and the outcome variables*. *This relationship will be stronger for employees working in governmental organizations as compared with those working in non-governmental organizations*.

**Fig 1:**
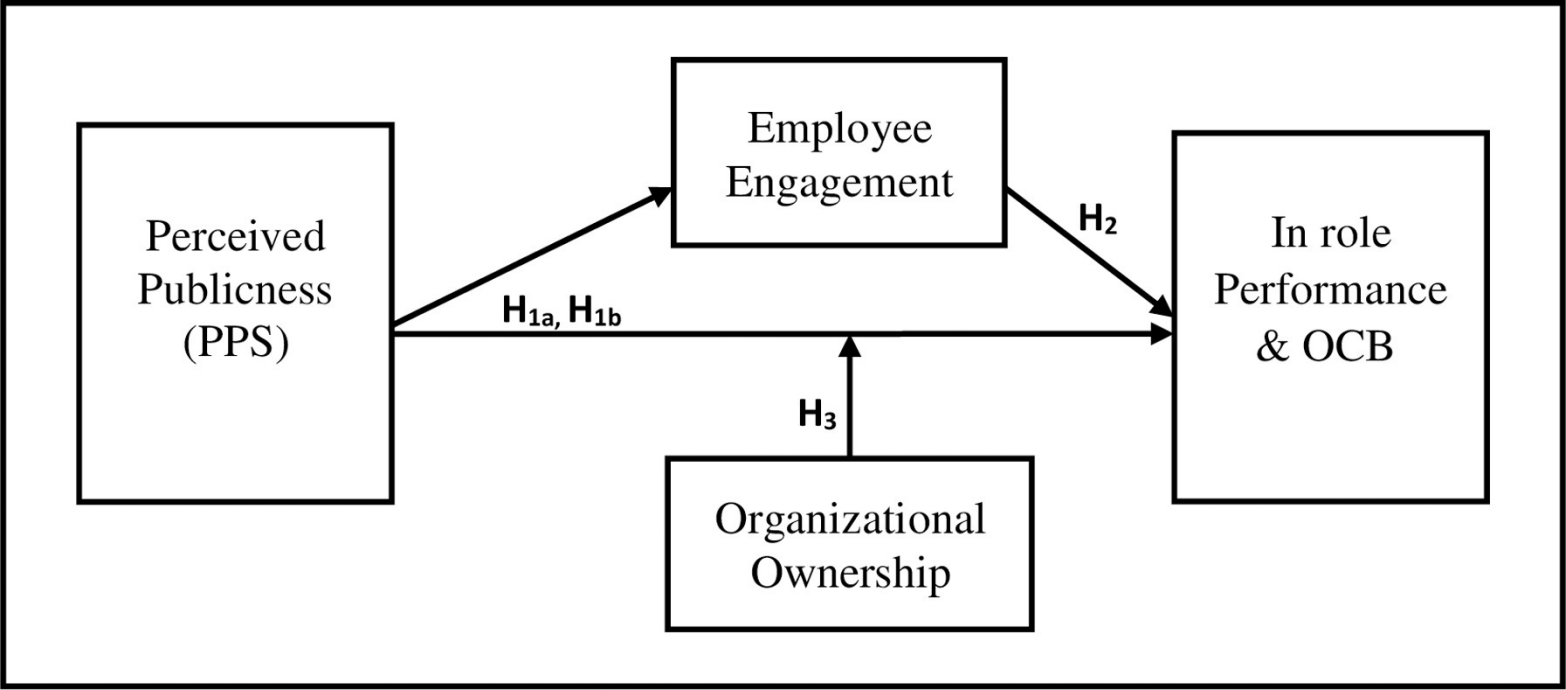
The research model.

Fig 1 summarizes our hypothesized model. It shows that perceived publicness is related to performance, measured by in-role performance and organizational citizenship behavior (OCB), both directly and indirectly through employees’ engagement as a mediator. In addition, the model includes organizational ownership as a moderator of these relationships.

## Method

### Sample and design

The study was approved by the Univ. of Haifa, Social Science Ethics Committee #071/17. To test our hypotheses, we conducted a study with a sample of 340 Israeli employees within four organizations. Employees’ consent was received in writing using a designated form and data was kept anonymous. Those who agreed to take part in the study were asked to complete a questionnaire that included items from the perceived publicness scale (PPS), an engagement scale, as well as questions relating to the control variables. The validation of the PPS was performed in two stages, using an international sample for the EFA analysis (N = 299), and using the current sample for the CFA analysis. The PPS was translated into Hebrew with the assistance of five expert reviewers. To reduce the potential for self-report and same-source bias, managers were asked to assess each of their participating employees’ level of in-role performance and OCB. Using two different data sources (e.g., managers and employees) was expected to strengthen the overall power of this study. Each employee was appointed a number to enable the researchers a way to match their completed questionnaires to those provided by their direct manager. We then matched the employee’s questionnaire to the manager’s questionnaire and eliminated any form of identification. The employees were either radiology technicians (n = 64) or security personnel (n = 276) from two government (n = 234) and two non-government (n = 106) organizations. The managers were also either radiology technicians (n = 2) or security personnel (n = 2) from the two government and the two non-government organizations. All managers were male with a mean age of 40.5 (S.D. = 17.5), and all hold an academic degree. We chose these professions because in Israel (a) both roles are regulated by the government, (b) employees across the sectors are expected to operate according to similar professional protocols when serving the public, and (c) each role has identical training and skill requirements regardless of organizational affiliation.

The overall response rate was 54%. The respondents were 76% males and 24% females (the imbalance derives from the fact that most security personnel are male). With regard to education, 82% of the respondents held an academic degree. The radiology technician respondents had a mean age of 32.7 (S.D. 10.26) and a mean tenure of 6.45 years (S.D. 7.53), and the security personnel had a mean age of 27.7 (S.D. 5.6) and a mean tenure of 3.53 years (S.D. 5.02). There were no significant gender, age, or tenure differences in the mean variables between the governmental and non-governmental organizations.

### Measures

*Perceived publicness* was measured using Dryzin-Amit et al.’s [25] 21-item PPS scale. Respondents provided answers on a 5-point Likert scale from 1 (not at all like my organization) to 5 (completely like my organization). Cronbach’s alpha for PPS was 0.93. *In-role performance and OCB* were measured using 14 items based on Williams and Anderson’s scale [67]. Respondents provided answers on a 5-point Likert scale from 1 (fully disagree) to 5 (fully agree). Cronbach’s alpha for in-role performance was 0.87 and for OCB was 0.92. As for *organizational ownership*, we categorized the organizations based on their ownership [68]: (1) core public organization, i.e. agencies owned by the government and funded with public money; (2) core private organizations, i.e. business firms and corporations owned by private individuals and funded through the sales of products or services with little or no funding from government contracts or hybrid organizations, which are service oriented professional organizations that deliver quasi-public goods and typically exist under both public and private ownership. Organizations were coded as governmental or non-governmental. *Employee Engagement* was measured by a 9-item scale based on the Utrecht Work Engagement Scale (UWES), which builds on Schaufeli et al. [16] and Balducci et al. [69]. In this case, we asked the respondents to indicate the degree to which they agreed with the statements on a 5-point Likert scale from 1 (fully disagree) to 5 (fully agree). Cronbach’s alpha for EE was 0.86.

Control Variables: We controlled for *gender*, *tenure and education* (1 = high school, 2 = professional accreditation, 3 = BA degree, 4 = MA degree or above). In addition, respondents provided additional information on: (1) *Transformational Leadership*, using a 15-item scale adopted from Rafferty and Griggin [70], Cronbach’s alpha for TFL was 0.93. (2) *Perceptions of Organizational Politics*, using a 5-item POP scale [71]. Cronbach’s alpha for POP was 0.84. We included these variables in our analysis to make sure that perceptions about publicness had an independent impact on the outcome variables, above and beyond any of the control variables. It is important to note that all Hebrew items and scales were based on established measures used in past studies (S1 Appendix) (e.g., PPS [25]; EE [59, 62]; In-role & OCB [72]; TFL [73]; PSM [74]; POP [75]).

### Analysis

Given that we used a multilevel design, i.e. with employees belonging to organizational units, we conducted a mixed model analysis using the SAS Mixed Model (MIXED) technique. Using multilevel modeling is essential for estimating factors that vary at more than one level of analysis. With the MIXED procedure we could test the random effect of the organizational unit and fit a random intercept model to allow for a different effect of the predictors on the outcome (OCB and in-role performance) across organizational units. This model takes into consideration the fact that individuals within one organizational unit might be more similar to one another than to individuals in other organizational units [76].

We tested all of the hypotheses based on a multilevel hierarchical method. The first model examined the effect of the demographic variables. The second model included also the other control variables. The third tested the main effects of perceived publicness. The fourth model investigated the role of organizational ownership as a moderator of the relationship between PPS and performance/OCB. Finally, the fifth model examined the effect of employee engagement as an initial indication of a mediation. To understand the nature of the significant moderating effects, we conducted a simple slope analysis [77]. To test the moderated-mediation effect we chose PROCESS modeling [78] and used a bias-corrected bootstrap assessment based on the approach of Preacher and Hayes (2008). This computationally intensive method involves repeated sampling from the data set and estimating the indirect effect in each resampled data set. Repeating this process thousands of times creates an empirical approximation of the sampling distribution that is then used to construct confidence intervals for the indirect effect [79]. Finally, we calculated the statistical significance of all of the effects, with a confidence interval of 95%, as MacKinnon, Lockwood, and Williams [80] suggested.

### Findings

Table 1 presents the means, standard deviations, and intercorrelations among the continuous study’s variables. Table 2 presents the distribution of PPS among the nominal variables. The bivariate findings in Table 1 indicate that as expected perceptions of publicness are positively related to employee engagement and OCB (r = .15; *p* < 0.01, and r = .20; *p* < 0.001, respectively) but not directly related to in-role performance (r = .06; p>.10). Interestingly there is a negative correlation between PPS and tenure (r = -.19; *p* < 0.001) indicating that less tenured employees perceive their organization as more public. Another interesting finding regards the positive significant correlations between leadership and PPS (r = .27, p < .001). This result indicates that employees that perceive their leader as highly transformative also perceive their organization as more public. While not the focus of the current study, it seems that employees that perceive their leaders as more visionary, providing personal consideration, invigorating intellectual challenges and serving as role models also perceive their organization as higher on publicness values. As found in previous research (e.g., [81]) significant correlations were found between in-role performance and OCB (r = .76; *p* < 0.001).

**Table 1:** Means, standard deviations, and intercorrelations.

	N	Mean	S.D.	1	2	3	4	5	6
1. Publicness Perceptions Scale (PPS)	340	3.74	.8	-	-	-	-	-	-
2. Tenure (years)	322	4.47	6.09	-.19***	-	-	-	-	-
3. Leadership	339	3.97	.73	.27***	-.23***	-	-	-	-
4. POP	340	2.41	.97	-.09	.20***	-.40***	-	-	-
5. Employee Engagement	337	3.46	.74	.15**	.12*	.37***	-.13*	-	-
6. In-role Performance	336	4.17	.63	.06	-.05	-.01	-.09	.10	-
7. OCB	339	3.86	.63	.20***	-.13*	.11*	-.14*	.16**	.76***

*p < .05

**p < .01

***p < .001

**Table 2:** Means and S.D. of perceived publicness across gender and education.

Variable	Categories	Sample Size			
			Mean	S.D.	F
Gender	Male	N = 260	3.70	.85	F(1,338) = 3.74
	Female	N = 80	3.90	.58	
Education	High School	N = 80	3.49	.58	F(3,332) = 9.18***
	Professional accreditation	N = 157	3.96	.82	
	BA degree	N = 30	3.82	.70	
	MA degree and above	N = 59	3.50	.87	

***p < .001

As can be seen in Table 2 while females perceive their organization on average as slightly higher on publicness, this difference was non-significant (F(1,338) = 3.74, p = .054), yet there was a significant difference in perceived publicness when examining differences in education (F(3,332) = 9.18, p < .001). A post hoc Tukey test revealed that people with professional accreditation perceive their organization as higher on the publicness scale as compared to those with high school education or those with MA degrees or above. Table 3 is especially interesting as it reveals that both type of organization (i.e. whether governmental or non-governmental) and profession type (i.e. whether radiologists or security personnel) have a significant impact on PPS. We found both significant main effects (F(1,336) = 22, p < .001 for profession and F(1,336) = 630.09, p < .001 for type of organization) and a significant interaction (F(1,336) = 53.70), with security personnel from the governmental organization reporting on average the highest level of PPS.

**Table 3:** Means and S.D. of perceived publicness across ownership and profession.

Sample Size	Profession	PPS Level in Government Organizations N = 234		PPS Level in Non-Government Organizations N = 106	
		Mean	S.D.	Mean	S.D.
N = 70	Security Personnel			2.58	.50
N = 36	Radiology Technicians			3.27	.36
N = 206	Security Personnel	4.21	.45		
N = 28	Radiology Technicians	3.79	.51		

F(3,336) = 235.26*** ***p < .001

H_1a_ suggested that perceived publicness will have a positive relationship with in-role performance. As Model 3 in Table 4 indicates, our data do not support this hypothesis. We found no direct relationship between perceived publicness and in-role performance (estimate = .06, p = .38). H_1b_ posited that perceived publicness will have a positive relationship with OCB. As Model 3 in Table 5 shows, in this case, the data supported our hypothesis. Perceived publicness had a positive relationship with OCB (estimate = .16; *p* < 0.01) above and beyond the control variables.

**Table 4:** Multi-level analysis with in-role performance as dependent variable.

	Model 1		Model 2		Model 3		Model 4		Model 5	
N	317		316		316		316		313	
	Estimate	S.E.	Estimate	S.E.	Estimate	S.E.	Estimate	S.E.	Estimate	S.E.
Intercept	4.20***	.14	4.40***	.31	4.18***	.40	3.85***	.49	4.07***	.45
Gender^+^	-.05	.08	-.04	.08	-.05	.08	-.06	.08	-.04	.09
Tenure	.01	.01	.01	.01	.01	.01	.01	.01	.00	.01
Education^++^	.01	.04	.00	.04	.01	.04	.01	.04	-.01	.04
Leadership			-.02	.06	-.02	.06	-.03	.06	-.04	.06
POP			-.05	.04	-.05	.04	-.04	.09	-.05	.04
PPS					.06	.07	.14	.09	.05	.08
Organization Ownership^+++^							1.04	.61	-.10	.22
PPS * Org. Ownership							-.35*	.18	-	-
Employee Engagement							-	-	.08	.05
Random Variance	.07	-	.07	-	.07	-	.06	-	.07	-
-2loglikelihood	314.2	-	620.3	-	623.0	-	621.8	-	612.4	

*p < 0.05,

** *p* < 0.01,

*** *p* < 0.001,

^+^1 = male, 2 = female;

^++^1 = high school, 2 = professional accreditation, 3 = BA degree, 4 = MA degree or above;

^+++^0 = non-government, 1 = government

**Table 5:** Multi-level analysis with OCB as dependent variable.

	Model 1		Model 2		Model 3		Model 4		Model 5	
N	320		319		319		319		316	
	Estimate	S.E.	Estimate	S.E.	Estimate	S.E.	Estimate	S.E.	Estimate	S.E.
Intercept	3.94	.14	3.81	.30	3.21	.38	3.43	.46	3.06***	.41
Gender^+^	-.09	.08	-.08	.08	-.08	.08	-.07	.08	-.07	.08
Tenure	.00	.01	.00	.01	.00	.01	.00	.01	.00	.01
Education^++^	.02	.04	.01	.04	.02	.04	.02	.04	.01	.04
Leadership	-	-	.05	.05	.03	.05	.03	.05	.01	.05
POP	-	-	-.02	.04	-.01	.04	-.02	.04	-.01	.04
PPS	-	-	-	-	.16**	.06	.11	.09	.14	.07
Org. Ownership^+++^	-	-	-	-	-	-	-.52	.58	-.08	.21
PPS * Org. Ownership	-	-	-	-	-	-	.14	.16	-	-
Employee Engagement	-	-	-	-	-	-	-	-	.11*	.05
Random Variance	.10	-	.10	-	.09	-	.10	-	.08	-
-2loglikelihood	568.1	-	564.6	-	558.8	-	558	-	233.9	-

*p < 0.05,

** *p* < 0.01,

*** *p* < 0.001,

^+^1 = male, 2 = female;

^++^1 = high school, 2 = professional accreditation, 3 = BA degree, 4 = MA degree or above;

^+++^0 = non-government, 1 = government

To test H_2_ that suggests that employee engagement will mediate the relationship between perceived publicness and the outcome variables, we analyzed the data using PROCESS, Model 4 [78]. The PROCESS procedure can be used to generate a bootstrap confidence interval for the indirect effects, which has become a widely recommended method for making inferences about mediation effects [78]. As Table 6 indicates, perceived publicness is related to employee engagement above and beyond the control variables (estimate = .14, p < .05). In addition, as Model 5 of Table 5 shows, employee engagement is related to OCB (estimate = .11, p < .05). No such relationship was found when in-role performance was the dependent variable (estimate = .08, p = .11). The analysis revealed that there is a mediated relationship when the outcome variable is OCB but not when the outcome is in-role performance. The 95% CI for OCB was (.001, .07) and did not include zero while for in-role it was (-.003,.025) including zero. Thus, the data supported H_2_ for OCB but not for in-role performance.

**Table 6:** Multi-level analysis with employee engagement as dependent variable.

	Employee Engagement			
	Model 1		Model 2	
N	317		317	
	Estimate	S.E.	Estimate	S.E.
Intercept	1.83***	.34	1.32**	.43
Gender^+^	.01	.09	.00	.09
Tenure	.03***	.01	.03***	.01
Education^++^	-.04	.05	-.03	.05
Leadership	.41***	.06	.39***	.06
POP	-.01	.04	-.01	.04
PPS	-	-	.14*	.07
Employee Engagement	-	-	-	-
Random Variance	.04	-	.05	-
-2loglikelihood	675.1	-	674.8	-

*p < 0.05,

** *p* < 0.01,

*** *p* < 0.001,

^+^1 = male, 2 = female;

^++^1 = high school, 2 = professional accreditation, 3 = BA degree, 4 = MA degree or above

**Fig 2:**
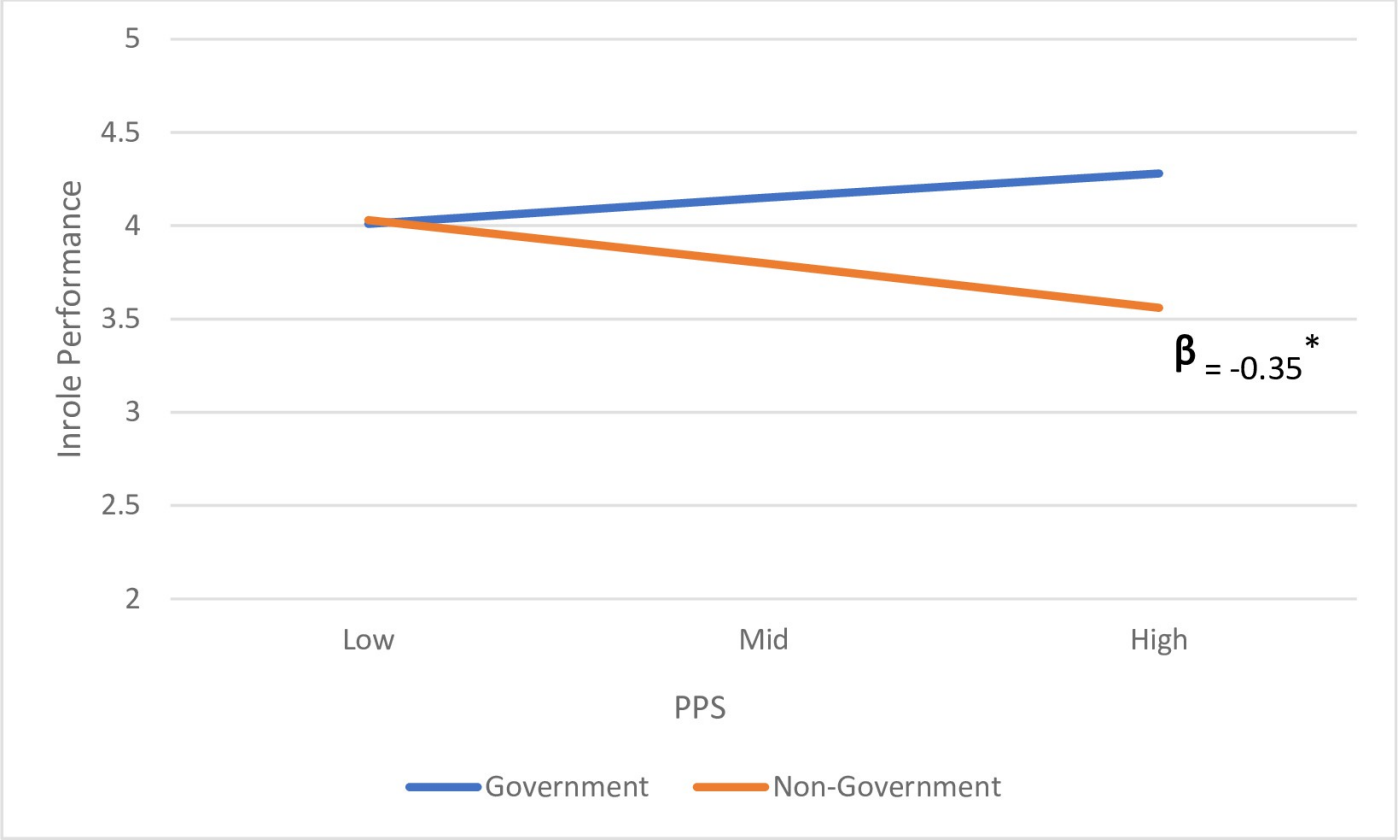
The relationship between perceived publicness and in-role performance.

Hypothesis H_3_ posited that organizational ownership will moderate the relationship between perceived publicness and the outcome variables, such that the relationship will be attenuated in non-government organizations. As Model 4 in Table 4 illustrates, the interaction of PPS*Org. ownership is significant (estimate = -0.35; *p* < 0.05). To understand the meaning of the interaction further, we plotted the simple slopes of perceived publicness on in-role performance in government vs. non-government organizations and tested their significance. As illustrated in Fig 2, the slope of in-role performance on perceived publicness is non-significant for government employees. However, the figure depicts a significant negative relationship for non-government employees (slope -.35; *p* < 0.05). Overall, these findings suggest that perceived publicness is negatively related to the in-role performance of employees in non-government organizations but does not affect their governmental counterparts. We found no such moderating effect when the dependent variable was OCB (estimate = .14, p = .39). Thus, the data support H_3_ for in-role performance but not for OCB. To sum, our findings indicate that when examining OCB, the relationship between perceptions of publicness and OCB is explained, at least partially by employee engagement. Yet when it comes to in-role performance, the relationship between publicness perceptions and in-role performance is not mediated by engagement but rather contingent on organization ownership. When the organization is not owed by the governmental perceptions of publicness are negatively related to performance.

To strengthen the above presented findings we conducted robustness checks and examined a number of additional models. First, we examined whether gender played a role in the relationship between PPS and our dependent variables. We ran three models with all variables included as well as the interaction between PPS and gender with OCB, in-role performance and employee engagement as dependent variables. The interaction between PPS and gender was not significant in any of these models indicating that the effects of PPS are not contingent on gender. In addition, as the sample of radiologists was small in comparison to the security personnel we examined all the above models on only the security personnel (n = 276). All the above findings were very similar when examining only this type of profession, minimizing the possibility that the uneven sample sizes was biasing the data. Finally, one could argue that the relationship between perceived publicness and OCB is the result of both constructs tapping employees’ altruism [8, 82]. To rule out this possibility and support our contention that perceived publicness is more than simply altruism, we measured altruism using Rushton, Chrisjohn, and Fekken’s [83] measure. This scale’s mean was M(346) = 3.6 with a S.D. = .6. The reliability coefficient of the scale was .60 and had a weak relationship with perceived publicness (r = .12; *p* < 0.05). When we added this variable as an additional control variable to Model 3 of Table 5, we found similar findings to those presented above without altruism.

## Discussion and summary

The purpose of this study was to provide a more comprehensive understanding of perceived publicness and its relationship with employees’ performance across organizations. Based on a variety of perspectives on differences between public and private organizations, and mainly using the neo-institutional rationality and perceptual approach to publicness we developed and tested a model and a set of hypotheses. Our major contribution is in relating publicness perceptions with employees’ formal and informal (i.e. OCB) performance. This contribution adds to current knowledge on publicness by (1) using a recently validated new scale of perceive publicness, (2) testing both formal and informal aspects of employees’ performance, as well as suggesting engagement as an important mediator, and (3) testing the cross-sector perspectives of these relationships.

The findings generally support many of the suggested relationships. Perceived publicness was found to have a positive relationship with employees’ OCB regardless of the organization’s ownership. This direct relationship between perceived publicness and OCB is aligned with Bozeman and Moulton’s [49] argument that publicness can best be managed through regulation in the public’s interest and based on ethics embedded in public values, regardless of sectorial affiliation. Critics of the public interest theory have argued that “*managing in the public interest presents no standard at all*, *but rather a declaration of intent”* ([8], p. 177). However, our findings may imply that publicness mainly relies on individual employees who are talented and committed to working in institutions that are guided by the ethos of serving the public interest. Thus, we suggest that perceived publicness may help find the tipping point where individual preferences about serving the public interest move organizations as institutions toward serving the public better, beyond simply complying with the formal requirements of governmental or non-governmental organizations.

In addition, we found support for the mediating effect of employees’ engagement in the relationship between perceived publicness and OCB. Employee engagement represents a positive, affective, motivational, work-related state of mind characterized by vigor, absorption, and dedication [16, 59]. Our findings support the assumption that perceived publicness can encompass several job resources including feeling valuable to others, feeling that life has meaning and purpose, gaining a sense of pride, hope, and an overall positive feeling about one`s role in society. These discrete positive emotions share the ability to broaden the employees’ momentary thoughts, actions, and repertoires and build their enduring personal resources, ranging from physical and intellectual resources to social and psychological resources [61]. Thus, when such crucial job resources are available, their level of engagement increases, improving their OCB.

Contrary to our expectations, we found no support for the direct or mediated effect of perceived publicness on in-role performance across organizations. Nevertheless, when organizational ownership was tested as a moderator, perceived publicness was negatively related to in-role performance of employees in non-government organizations but not to in-role performance of governmental employees. One interpretation of this finding is that in non-governmental organizations, a gap between employees’ views of publicness and the organizational identity as a private or hybrid one, may result with lower formal performance. On the other hand, publicness perceptions do not seem to have an effect in governmental organizations where this gap is much smaller. Thus, and in accordance with person-organization fit theories (e.g., [84, 85]) the value based fit between individuals and organizations may be important for fulfilling one’s in-role performance [86].

What is the meaning of our findings for government, administration, and organization studies? The literature in the field has extended ([6]) and pointed to publicness as a highly relevant theme due to several reasons: (1) the expansion of the hybrid sector (e.g., [87]); (2) the blurring between the sectors [30]; (3) the trend to create "Shared Value" (e.g., [88]) and implement "Corporate Social Responsibility" standards in the private sector (e.g., [89]); (4) the expectations of Millennials to increase prosocial and societal impact on the job (e.g., [13]); (5) the growing market orientation of public service organizations ([8]); and (6) the influence of political populism on public service provision [90]. We believe that our study and its findings not only provide a way to understand publicness in a more lucid manner that extends the above studies, but to show that perceptions of publicness play an important role regardless of organizational affiliation. Thus, future research may wish to include such perceptions in theory and empirical studies.

Our study is also one of a sequence that adheres with the trend of elevating publicness theory to the perceptual level (e.g., [7, 50, 91]). We used perceived publicness as a valid measure of publicness that may broaden our view and open up a new avenue for the exploration of performance in governmental and non-governmental organizations. Our approach tried to provide a fuller spectrum of publicness dimensions and to contribute to the understanding of employees’ performance in governmental and non-governmental organizations. By doing so we added to the knowledge in public administration and management, general business and management literature, and generic human behavior literatures. Regardless of whether an organization is defined as being owned by a private, public or hybrid entity, employees who regard the organization as more concerned with the public engaged in more OCB. Furthermore, demonstrating an indirect link between publicness and OCB through engagement as a mediator may open up an additional avenue for theory and practice. It indicates that values, purpose, and attentiveness to the public are related to the extent to which an employee feels connected to his/her organization which in turn relates to OCB across sectors. Thus, theories of engagement and OCB may wish to include elements of publicness. Moreover, implications for human resource management practices may be drawn based on such theories.

In addition, we also established that the lack of fit between an employee’s perceptions about publicness and those of the organization may hamper employee’s in-role performance and duty-related behaviors. Documenting this factor adds an additional element to theories about the need for a fit between the individual and the organization [84]. While most theories in this area consider this fit with regard to shared values, organizational ownership also seems to play a role. More specifically, in non-governmental organizations, when there is a poor fit (i.e. when employees perceive the organization’s publicness as high), implications are drawn in terms of employees’ performance. Thus, promoting perceived publicness as a framework to define employees’ role in relation to customers, citizens, politicians, and organizations may be problematic and be negatively related to performance at the individual level, at least in some organizations.

### Limitations and future research

Despite its advantages, this study has several limitations. First, participants were sampled in Israel, hence replication in other national contexts is needed to extend generalization of the findings. Second, it was not possible to sample private employees serving in roles identical to those held by their hybrid or public sector counterparts. Hence, future studies should further validate the perceived publicness scale using samples of private sector employees. Third, we tested the perceived publicness scale by sampling two major professions (radiology technicians and security personnel). Testing the predictive power of the PPS among other professions would highly contribute to the validation of our findings. In addition, many of our variables were measured using employees’ self-reports which may be subject to some bias. However, as the dependent variables were rated by the employee’s managers there is less of a worry of common method bias. To ensure this, we further conducted Harman’s single factor test with all items in the employee’s survey (i.e. items for the leadership, POP, EE and PPS scales). We followed Podsakoff et al. [15] and examined the un-rotated factor solution to determine the number of factors that are necessary to account for the variance in the variables. In accordance with our model and design, the un-rotated factor solution resulted with 10 factors, with the factor with the highest Eigenvalue accounting for only 25% of the variance. This minimizes the likelihood of potential bias in our study.

Finally, we based our arguments, rationale, and hypotheses on the neo-institutional theory [17, 19]. However, we acknowledge that other theories may also be very useful in dealing with studies about perceptions of publicness. Examples include theories about social identity and person-organization fit [92], self-affirmation [93], attraction-selection-attrition [94] and exit, voice, and loyalty [95]. These theories may be very useful for future explorations about the relationships between the intra-factors of publicness and outcome variables such as performance.

Based on our study’s theory and findings additional important question should be discussed and examined in future studies. An exemplary battery of such studies and questions may include: (1) should governments define what belongs to the public or private sphere given these perceptions of these spheres vary between individuals (2) how may perceptions of publicness affect other organizational or individual level factors? and, (3) how can organizations better use publicness perceptions to provide more equality public outcomes?

## Conclusions

Although the publicness enigma may have not been fully resolved, we believe that this study made a significant step forward in clarifying some of its aspects, especially in regard to employees’ performance across sectors. We tried to advance the scholarly effort regarding questions and conceptual inconsistencies related with the theoretical meaning of publicness for different stakeholders and organizations, as well as for its relationship with employees’ performance. We generally found that perceived publicness matters for both the formal and informal aspects of employees’ performance and that, engagement and sector are important variables that play mediating and moderating roles in these relationships. Thus, our study quite strongly supports the neo-institutional theory and the perceptual line of thinking on publicness and performance.

Beyond the theoretical merit our results provide several practical contributions: First, the publicness perceptions approach and scale may help to develop and conduct practical interventions to advance understanding of public related duties among employees. Such interventions may increase awareness amongst employees and managers regarding how their work influences society and what public and publicness means in their daily organizational life [12, 13], thus helping improve the organization’s ability to effectively provide public goods and services. In addition, clearer use of the publicness perception scale may assist managers in identifying highly public oriented individuals; increase the level of fit between employees and organizations [93], and be used when developing organizational strategies for useful interfaces between the organization and its external stakeholders and the public.

All in all, we believe that this study contributes in better acknowledging the theoretical and practical role of publicness perceptions in organizational dynamics in both government and non-government organizations.

## Supporting information

S1 Data (XLS)

S1 Appendix (DOCX)
