# Supplementary material for: The publicness enigma: Can perceived publicness predict employees’ formal and prosocial behavior across sectors?
Source: PLoS One. 2022 Feb 10;17(2):e0262253. doi: 10.1371/journal.pone.0262253 (PMC8830704; doi:10.1371/journal.pone.0262253)
Supplement: S1 Appendix — (DOCX) [file pone.0262253.s002.docx]

# **Supporting Information**

**S1 - Data file**

# **S2- Appendix 1: Scales Items**

## **Publicness Perception Scale (PPS)**

Ownership

1. The organization where I work is owned by the state.
2. The organization where I work is public by law.
3. I see myself as a civil servant.
4. The organization where I work is a public organization.

Source of Funding

1. Our organization’s budget is set by the government or the local authority.
2. As far as I know, my salary is paid by the state or the local authority and not from any private sources.
3. My salary is funded by taxes the state collects from citizens.
4. The government is the one that decides about the budget of my organization.

Political Control

1. The government or local authority exercises a great deal of supervision over the operations of our organization.
2. The main purposes of the organization where I work are set by the government, and we as employees of this organization can't change them.

Market Control

1. Performance in my organization cannot be assessed by a financial " bottom line."
2. Economic measures cannot fully evaluate performance in my organization.

Legality

1. In general, all citizens have a basic right to be served by us.
2. In the organization I work for, citizens have a right to be served by us.
3. The organization where I work makes an effort to adjust its services to the special needs of all the members of our society.

Transparency

1. The organization I work for is responsive to the freedom of information law.
2. In the organization I work for, we conform to most requests for information from the public or others openly.

Accountability

1. In the organization I work for, failures or mistakes tend to draw public attention.
2. The public expects my organization to manage its services responsibly.

Equity

1. Those who are politically connected do not receive preferential treatment in my organization.
2. Members of the upper class of society do not receive preferential treatment in my organization.

## **Employee Engagement Scale**

Facets of Employee Engagement in the Public Sector: (Schaufeli et al., 2002)

Vigor

1. At my work, I feel bursting with energy.

2. At my job, I feel strong and vigorous.

3. When I get up in the morning, I feel like going to work.

Dedication

1. To me, my job is challenging.

2. My job inspires me.

3. I find the work that I do full of meaning and purpose.

Absorption

1. It is difficult to detach myself from my job.

2. I feel happy when I am working intensely.

3. Time flies when I`m working.

## **OCB & In-role Performance Scale**

Facets of OCB and in-role performance (Williams & Anderson, 1991). Managers indicated how much they agree that the specific employee performs this behavior.

In-role performance:

1. Adequately completes assigned duties.
2. Fulfills responsibilities specified in job description.
3. Performs tasks that are expected of him/her.
4. Meets formal performance requirements of the job.
5. Engages in activities that will directly affects his/her performance evaluation.
6. Neglects aspects of the job he/she is obligated to perform. (R)
7. Fails to perform essential duties. (R)

OCB:

1. Helps others who have been absent.
2. Helps others who have heavy workloads.
3. Assists supervisor with his/her work (when not asked).
4. Takes time to listen to co-workers` problems and worries.
5. Goes out of way to help new employees.
6. Takes a personal interest in other employees.
7. Passes along information to co-workers.
8. Attendance at work is above the norm.
9. Gives advance notice when unable to come to work.
10. Takes undeserved work breaks. (R)
11. Great deal of time spent with personal phone conversations. (R)
12. Complains about insignificant things at work. (R)
13. Conserves and protects organizational property.
14. Adheres to informal rules devised to maintain order.

**Perceptions of Politics (POP)** (Kacmar & Carlson, 1997)

1. There is no place for yes-men around here: good ideas are desired even if it means disagreeing with supervisors (R)
2. People in this organization attempt to build themselves up by tearing others down.
3. When it comes to pay and promotion decisions policies are irrelevant
4. There has always been an influential group in this organization that no one ever crosses.
5. Agreeing with powerful others is the best alternative in this organization.

**Transformational Leadership (TFL) (**Rafferty & Griggin, 2004)

Vision

1. Has a clear understanding of where we are going.

2. Has a clear sense of where he/she wants our unit to be in 5 years.

3. Has no idea where the organization is going (R)

Inspirational communication

4. Says things that make employees proud to be a part of this organization.

5.. Says positive things about the work unit.

6.. Encourages people to see changing environments as situations full of opportunities.

Intellectual stimulation

7. Challenges me to think about old problems in new ways.

8. Has ideas that have forced me to rethink some things that I have never questioned before.

9. Has challenged me to rethink some of my basic assumptions about my work.

Supportive leadership

10. Considers my personal feelings before acting.

11. Behaves in a manner which is thoughtful of my personal needs.

12. Sees that the interests of employees are given due consideration.

Personal recognition

13. Commends me when I do a better than average job.

14. Acknowledges improvement in my quality of work.

15. Personally compliments me when I do outstanding work.
